# Complete Genome Sequence of the Silicimonas algicola Type Strain, a Representative of the Marine *Roseobacter* Group Isolated from the Cell Surface of the Marine Diatom Thalassiosira delicatula

**DOI:** 10.1128/MRA.00108-19

**Published:** 2019-02-28

**Authors:** Klervi Crenn, Boyke Bunk, Cathrin Spröer, Jörg Overmann, Christian Jeanthon

**Affiliations:** a CNRS & Sorbonne Université, Station Biologique de Roscoff, Adaptation et Diversité en Milieu Marin, Team ECOMAP-Ecology of Marine Plankton, Roscoff, France; b Leibniz Institute DSMZ—German Collection of Microorganisms and Cell Cultures, Braunschweig, Germany; c Microbiology, Braunschweig University of Technology, Braunschweig, Germany; University of Southern California

## Abstract

Silicimonas algicola strain KC90B^T^ is an alphaproteobacterium of the *Roseobacter* clade that was isolated from a culture of the marine diatom *Thalassiosira delicatula*. Here, we report the complete genome sequence of this type strain, which is 4,351,658 bp in size with 4,272 coding sequences and an average G+C content of 65.2%.

## ANNOUNCEMENT

Members of the *Roseobacter* clade (*Alphaproteobacteria*) are often dominant in natural assemblages with marine algae and are often found in laboratory cultures of marine phytoplankton (1–3). Silicimonas algicola strain KC90B^T^ (= DSM 103371T = RCC 4681^T^) was isolated from the cell surface of the marine diatom Thalassiosira delicatula RCC 2560 (4) in July 2013. This microalgal culture isolated from the coastal long-term monitoring station SOMLIT-Astan (north of Roscoff, France, in the western English Channel) has been maintained in the RCC since its isolation in January 2011.

Cells were grown in modified marine broth (2.5 g peptone, 0.5 g yeast extract, and 35 g sea salts dissolved in 1 liter of Milli-Q water) at 20°C, and 500 mg was harvested after 15 days. Genomic DNA was isolated using Qiagen Genomic-tip 100/G (Qiagen, Hilden, Germany) according to the manufacturer’s instructions. For long read sequencing, a SMRTbell template library was prepared following the procedure and checklist—greater than 10 kb template preparation (Pacific Biosciences, Menlo Park, USA). Briefly, 8 µg genomic DNA was sheared for preparation of 15-kb libraries. DNA was end-repaired and ligated overnight to hairpin adapters applying components from the DNA/polymerase binding kit P6 (Pacific Biosciences). BluePippin size selection to greater than 4 kb was performed (Sage Science, Beverly, USA). Conditions for annealing of sequencing primers and binding of polymerase to a purified SMRTbell template were assessed with the calculator in RS Remote (Pacific Biosciences). Single-molecule real-time (SMRT) sequencing was carried out on the PacBio RS II system (Pacific Biosciences). SMRT sequencing revealed a total of 69,135 reads with a mean read length of 14,050 bp and an *N*_50_ value of 17,953 bp. From the same batch of DNA, a short insert library was created using the NEBNext ultra DNA library prep kit for Illumina (NEB, Ipswich, USA). Short-read sequencing was carried out on an Illumina HiSeq 2500 platform resulting in 4,006,700 paired-end reads of 2 × 101 bp.

Genome assembly was performed by applying the Hierarchical Genome Assembly Process version 3 (RS_HGAP3) protocol included in SMRT Portal 2.3.0 using default parameters. The assembly revealed a single circular chromosome with a coverage of 146×. The chromosome was circularized, and artificial redundancies at the ends of the contigs were removed and adjusted to *dnaA* as the first gene. Error correction was performed by a mapping of Illumina short reads onto the finished genome sequence using Burrows-Wheeler Alignment (BWA) 0.6.2 in paired-end (sample) mode using default settings (5) with subsequent variant and consensus calling using VarScan 2.3.6 (6). A consensus concordance of quality value (QV) 60 was reached. Automated genome annotation was carried out using the NCBI Prokaryotic Genome Annotation Pipeline (PGAP) (7).

The complete genome sequence of Silicimonas algicola strain KC90B^T^ consists of a single circular chromosome with 4,351,658 bp and a G+C content of 65.2%. The NCBI PGAP predicted 4,272 coding sequences, 42 tRNA genes, and 1 rRNA operon. The genome contains biogeochemically relevant pathways previously reported for roseobacters, such as carbon monoxide oxidation (*cox*), sulfur oxidation (*sox*), and phosphonate utilization (*phn*) genes (8). It also shows a prevalence of genes involved in environmental stress response and detoxification, tripartite ATP-independent periplasmic (TRAP) and ABC transporters, and carbohydrate- and electron-accepting reactions. The genome annotation also revealed the presence of quorum-sensing genes, such as an *N*-acyl-l-homoserine lactone synthetase (*luxI*) and a regulator protein belonging to the *luxR* family on the same operon (9).

### Data availability.

The complete genome sequence of S. algicola strain KC90^T^ (= DSM 103371T = RCC 4681^T^) has been deposited in the NCBI GenBank under the accession no. CP034588 (BioProject no. PRJNA504651, BioSample no. SAMN10396653). The version described in this paper is the first version, CP034588.1. Raw sequence reads have been submitted to the NCBI SRA under the accession no. SRR8529664 (PacBio) and SRR8529665 (Illumina).
